# Energy Metabolic Plasticity of Colorectal Cancer Cells as a Determinant of Tumor Growth and Metastasis

**DOI:** 10.3389/fonc.2021.698951

**Published:** 2021-07-26

**Authors:** Leenu Reinsalu, Marju Puurand, Vladimir Chekulayev, Sten Miller, Igor Shevchuk, Kersti Tepp, Egle Rebane-Klemm, Natalja Timohhina, Anton Terasmaa, Tuuli Kaambre

**Affiliations:** ^1^ Laboratory of Chemical Biology, National Institute of Chemical Physics and Biophysics, Tallinn, Estonia; ^2^ Department of Chemistry and Biotechnology, School of Science, Tallinn University of Technology, Tallinn, Estonia

**Keywords:** tumor energy metabolism, aerobic glycolysis, oxidative phosphorylation, VDAC, creatine kinase, adenylate kinase, mitochondria

## Abstract

Metabolic plasticity is the ability of the cell to adjust its metabolism to changes in environmental conditions. Increased metabolic plasticity is a defining characteristic of cancer cells, which gives them the advantage of survival and a higher proliferative capacity. Here we review some functional features of metabolic plasticity of colorectal cancer cells (CRC). Metabolic plasticity is characterized by changes in adenine nucleotide transport across the outer mitochondrial membrane. Voltage-dependent anion channel (VDAC) is the main protein involved in the transport of adenine nucleotides, and its regulation is impaired in CRC cells. Apparent affinity for ADP is a functional parameter that characterizes VDAC permeability and provides an integrated assessment of cell metabolic state. VDAC permeability can be adjusted *via* its interactions with other proteins, such as hexokinase and tubulin. Also, the redox conditions inside a cancer cell may alter VDAC function, resulting in enhanced metabolic plasticity. In addition, a cancer cell shows reprogrammed energy transfer circuits such as adenylate kinase (AK) and creatine kinase (CK) pathway. Knowledge of the mechanism of metabolic plasticity will improve our understanding of colorectal carcinogenesis.

## Introduction

Analysis of mitochondrial function is central to the study of intracellular energy metabolism and pathophysiological mechanisms of various human diseases, including cancer. The metabolism of cancer cells is adapted to meet their needs to survive and proliferate in a hypoxic and also in a well-oxygenated microenvironment and thus must acquire metabolic flexibility. At the molecular level, metabolic flexibility relies on the configuration of metabolic pathways, which are regulated by key metabolic enzymes and transcription factors. Reprogramming of cellular energetics is recognized as a distinctive hallmark of cancer (1). The first theory on the peculiarities of cancer metabolism was formulated by Otto Warburg in the early 20th century. He concluded that tumors, unlike normal cells, obtain their energy mainly from aerobic glycolysis, while normal cells usually favor oxidative phosphorylation (OXPHOS), which is much more efficient in terms of ATP gain. This observation is coined as the Warburg effect (2, 3) and became the central model for oncobioenergetics for most of the 20^th^ century. The glycolytic part of the Warburg hypothesis was firmly and thoroughly confirmed for many cancer types, in contrast to the OXPHOS part, which was and still is a matter of intense research and controversy. Verified evidence indicates that in reality, both anaerobic (glucose to lactate) and aerobic (glucose to pyruvate) glycolysis operate in cancer cells simultaneously like in normal cells, although at higher rates than in non-tumor cells (4). In addition, tumor cells often exhibit high rates of OXPHOS (5, 6). Transcriptomics and end-product metabolites analyses of complex molecular pathways converge into a three-node minimum regulatory network consisting of hypoxia-inducible factor 1 (HIF-1), adenosine monophosphate-activated protein kinase (AMPK), and reactive oxygen species (ROS). Therefore, the coexistence of three distinct cellular metabolic phenotypes is revealed in cancer cells: 1) glycolytic, characterized by high activity of HIF-1α and high activity of the glycolytic pathway; 2) OXPHOS state, characterized by high activity of AMPK and high activity of OXPHOS pathways such as glucose oxidation and fatty acid oxidation; 3) hybrid metabolic state, characterized by high activity of AMPK and HIF-1α and concomitant functioning of glycolysis and OXPHOS pathways. In contrast, normal cells exhibit only two metabolic states, namely, glycolytic and OXPHOS, and lack the hybrid state (7, 8). In this regulatory network, HIF-1 and AMPK are the master regulators of glycolysis and OXPHOS, respectively (9), and both cytosolic and mitochondrial ROS mediate the complex interplay between AMPK and HIF-1. Accordingly, the hybrid metabolic state in cancer cells can be promoted by the stabilization of HIF-1α and elevated production of mitochondrial ROS. Hypoxia activates glycolysis *via* stabilization of HIF-1α and HIF-2α, which in turn upregulates the activity of several members of the glycolytic pathway and increases glucose uptake (10, 11). In addition, the elevation of HIF-1α levels could be induced by high concentrations of succinate (pseudohypoxia) (12). A striking feature of cancer cells is their ability to switch their metabolic phenotypes to glycolysis or OXPHOS in response to changes in their microenvironment or inhibition of one of these pathways, giving survival advantage during tumor progression (8, 13). This metabolic plasticity is promoted by the hybrid phenotype of cancer cells and is linked with metastasis and chemoresistance (14). However, it is still largely unknown how cancer cells regulate gene expression to maintain their hybrid metabolic state and metabolic plasticity.

Implementation of the hybrid metabolism paradigm may reveal new therapeutic targets and opportunities for the treatment of cancer. It was previously shown that administration of glycolytic inhibitors alone may be ineffective to eradicate tumors, and targeting the hybrid state to eliminate metabolic plasticity could be a new therapeutic strategy to eliminate cancer aggressiveness (15, 16). We review the changes in OMM permeability and intracellular energy transfer pathways in connection with the metabolic plasticity of CRC cells.

## Metabolic Reprogramming of Colorectal Cancer

Colorectal cancer has been regarded as a purely hypoxic tumor of the Warburg phenotype for many years. This was confirmed by increased expression of several glycolytic enzymes, pentose phosphate pathway, and glucose transporters associated with elevated rates of glucose consumption and lactate production as compared with normal surrounding tissues (17–25). Normal colonocytes use the OXPHOS system as the primary energy source (26, 27). Short-chain fatty acids undergo β-oxidation to form acetyl-CoA, which enters into the tricarboxylic acid (TCA) cycle to yield citrate, NADH, and finally ATP. But, unlike normal colonocytes, colorectal carcinomas cannot utilize butyrate as an energy source and carbon donor (26, 28), implying the truncated TCA cycle in CRC. Importantly, some metabolites of the TCA cycle, such as succinate, fumarate, and α-ketoglutarate, act as “oncometabolites” that support tumor growth *via* oncogenic signaling, inter alia *via* upregulation and stabilization of HIF-1α (29).

Metabolic reprogramming during large intestine carcinogenesis is largely mediated by (a) altered expression of several oncogenes and a loss of tumor suppressor genes, encoding usually various transcriptional factors and protein kinases (30, 31), (b) adaptation to nutrient and oxygen availability in the local tumor microenvironment (metabolic plasticity) (32), and (c) metabolic cross-talk with stromal, adipose tissue and immune cells (31, 33–37).

Data on molecular mechanisms of the metabolic reprogramming of CRC are mostly obtained from studies using cell culture models, while the number of functional studies using clinical material is limited. Moreover, cell culture conditions have variations that could significantly affect the metabolic profile of the cells. For example, cells grown in glucose-free medium display a relatively high rate of oxygen consumption, while cultivation of cells in a high-glucose medium results in hyperglycolytic profile and declined respiratory flux (38–42). Our recent studies revealed remarkable differences in the regulation of outer mitochondrial membrane (OMM) permeability between cultured tumor cells and clinical material from cancer patients (5, 43). Comparative analysis of the biopsy or surgical cancer material and surrounding healthy tissue showed almost unchanged glycolytic activity and upregulation of OXPHOS in CRC, which is inconsistent with the data obtained by using cell culture (43–47). In addition, two widely used breast cancer cell lines MCF7 and MCF-MDA-231 failed to replicate mitochondrial function in respect to metabolic activity and OXPHOS as seen in respective human samples (43, 46).

Why the CRC cells shift their metabolism in favor of OXPHOS? Perhaps, under normal conditions, the amount of ATP produced through aerobic glycolysis is insufficient to support cell proliferation and migration. There is a growing body of evidence that CRC is characterized by stimulated mitochondrial biogenesis expressed as an increase in mitochondrial DNA copy number (48) and elevated ADP-dependent oxygen consumption in CRC tissue (5, 6, 43–45). Activated mitochondrial biogenesis can be an adaptive response of tumor cells to overcome the chronic energy crisis caused by glucose starvation or defects in the function of their respiratory enzymes due to pathogenic nuclear or mtDNA mutations (49–51). The elevated lactate level may act as a signaling molecule to affect genes and proteins known to be involved in mitochondrial biogenesis (52), *via* upregulation of AMPK- and SIRT1-associated PGC-1α activation (53). Nuclear Respiratory Factor 1 (NRF1) (54) and some cytokines, IL-6/8 (55, 56), activate the AMPK signaling pathway as well as apoptotic resistance of cancer cells (56–58). Some types of tumor cells support their high rates of OXPHOS and drug resistance by transferring mtDNA or even the entire mitochondria from surrounding healthy tissues; this intercellular mitochondrial transfer may occur through exosomes or tunnel nanotubes (59, 60). The signaling pathways responsible for the stimulation of mitochondrial biogenesis can have both intracellular and external origins.

## The Role of VDAC and the Regulation of Outer Mitochondrial Membrane Permeability in Metabolic Plasticity

The flux of water-soluble metabolites into and out of the mitochondria occurs through a variety of inner mitochondrial membrane (IMM) carriers, but the flux of ATP, ADP, and Pi across the OMM occurs through a single pathway, the VDAC, and therefore the regulation of OXPHOS is largely mediated by the VDAC permeability control (61). Based on studies of muscle permeabilized fibers, cellular respiration and associated ATP synthesis are regulated by a protein complex called Mitochondrial Interactosome (MI), which is located at the junction of mitochondrial membranes (62, 63). Restrictions for adenine nucleotides in VDAC are evident by measuring an apparent affinity of mitochondria for exogenous ADP [Km(ADP)] in permeabilized cells and tissues by using high-resolution respirometry (64, 65). These barriers appear only in permeabilized cells and not in isolated mitochondria and disappear during mild proteolytic treatment with trypsin (66). Therefore, the metabolic plasticity of cancer cells is associated with the protein-mediated control of VDAC permeability towards ADP.

### Cancer Metabolic Plasticity Is Functionally Defined by Changes in ADP Dependent Oxygen Consumption

**Figure 1:**
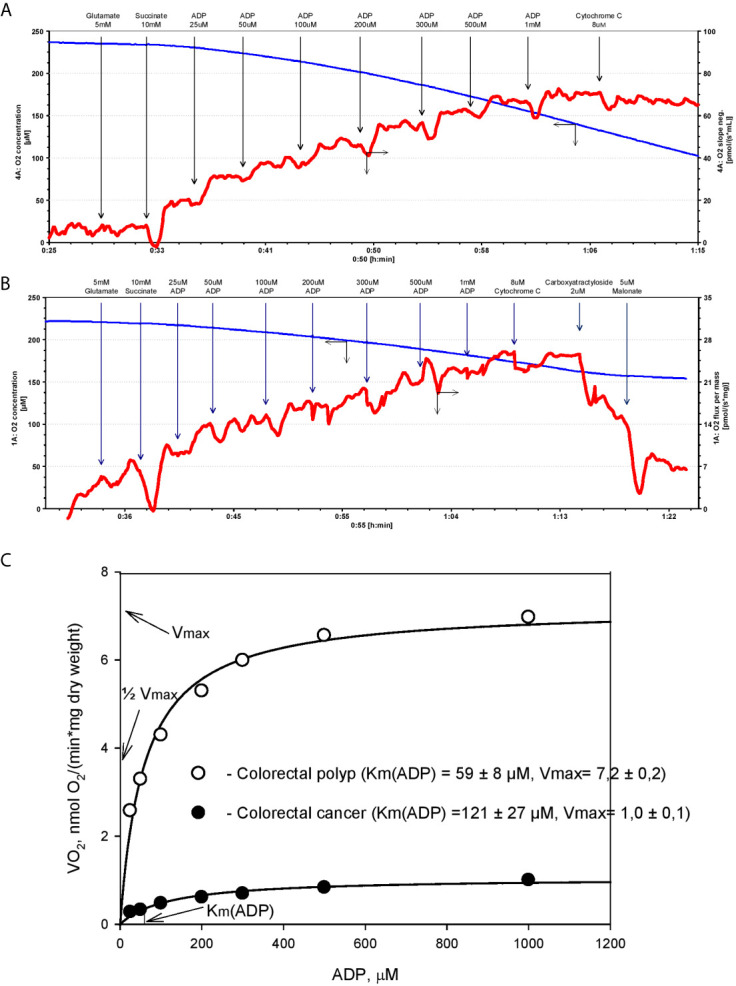
Michaels-Menten kinetics of ADP-dependent respiration of human colorectal cancer and polyp biopsy material. Representative tracing of adenosine diphosphate (ADP)-activated oxygen consumptions rates in human permeabilized tissue of **(A)** colorectal polyp and **(B)** and colorectal cancer. **(C)** Corresponding Km(ADP) and V_max_ values were calculated by non-linear regression using the Michaelis-Menten equation.

Analysis of respirometry data provides instant functional profiling of metabolic plasticity. Dependence of mitochondrial O_2_ consumption upon ADP concentration follows Michaelis-Menten kinetics and allows evaluation of apparent Michaelis-Menten constant for ADP Km(ADP) in different tissues, cancers, and cell cultures (**Figure 1**). Determined in permeabilized cells and tissues, Km(ADP) is the affinity of the mitochondria for exogenous ADP and characterizes permeability of OMM for adenine nucleotides and, thus, VDAC permeability. Measured Km(ADP) values for human colon mucosa is ~110 µM (47), ~100 µM for CRC (5, 44, 47), ~60 µM for colon polyps (47), and ~40 µM for Caco2 CRC cell line (43), indicating the alteration of control mechanisms over VDAC permeability and OXPHOS during the progression of CRC. Thus, the regulation of OMM permeability to adenine nucleotides in cancer tissues is different from that in normal cells (5, 67, 68). Notably, Km(ADP) values measured in cell cultures are much lower than in tissue biopsies and are similar to Km(ADP) values for isolated mitochondria (69). This illustrates the shortcomings of cell culture studies and highlights the importance of using clinical material for the evaluation of the mechanism of cancer metabolic plasticity.

The cell-specific differences in Km(ADP) are likely caused by the specific structural and functional organization of energy metabolism. For example, cells with a low Km(ADP) value (~10 µM), like glycolytic muscle, possess less structural and functional restrictions for ADP/ATP movement through OMM as compared to the oxidative muscles (Km(ADP) ~300 µM) (64). Thus, relatively low Km(ADP) for colorectal polyps indicates a metabolic reprogramming towards the glycolytic phenotype with functional OXPHOS (as in glycolytic muscle), and an increase in Km values in the CRC reflects a shift to OXPHOS phenotype with increased intracellular complexity (analogy with oxidative muscle). Hence, Km(ADP) value is an important parameter describing metabolic plasticity. According to the model proposed by Saks V. et al, the proportion of mitochondria with low oxidative capacity in the tissue can be inferred from the Km(ADP) value (70). For example, the proportion of mitochondria with high oxidative capacity is 67% in CRC tumors and only 38% in colorectal polyps (47).

In addition to Km(ADP), the maximal ADP-dependent oxygen consumption (V_max_) is a defining characteristic of metabolic plasticity and is correlated to mitochondrial content (density) in the tissue. V_max_ values are higher in CRC than in normal colon tissue (5, 6, 47), indicating a vigorous metabolic activity. Moreover, V_max_ values in biopsy material from patients that succumbed to colon cancer were significantly higher than in patients staying in remission (5). However, the extent to which high V_max_ values correlate with tumor aggressiveness needs to be confirmed in further studies.

### The Possible Mechanisms of VDAC Permeability Regulation

Several studies show that VDAC isoform 1 (VDAC1) is the dominant isoform in most malignant tumors including CRC (44, 71, 72). VDAC1 is crucial in communication between the mitochondria and the cytosol. Cancer cells display high levels of metabolic flexibility combined with apoptosis resistance, which provides a survival advantage for these cells. VDAC1 is well recognized as a metabolic checkpoint at the crossroad of these two processes (72, 73). VDAC mediates and regulates the transport of metabolites, ions, and ROS across OMM. Thus, VDAC1 plays a major role in the control of mitochondrial function. Transport of ADP through OMM is mediated *via* VDAC1 and through the inner membrane *via* ANT. Metabolic control analysis of the OXPHOS system of CRC revealed that ANT does not exert exclusive control over the mitochondrial ADP-dependent oxygen consumption (5, 43). Therefore, the rate-limiting step of ADP transport into the mitochondria appears to be VDAC. Therefore, the alteration of Km(ADP) value depends on the changes in interactions of VDAC1 with other proteins or on the modification of VDAC1 itself.

As the name implies, VDAC is regulated by a change of membrane potential. Studies of isolated VDAC1 reconstituted into planar lipid bilayers reveal sharp and symmetrical voltage dependence of VDAC1 permeability (72, 74, 75). At membrane potentials close to zero (between −20 to +20 mV), VDAC1 is open and displays low anionic selectivity. At more positive or more negative membrane potentials (+30.+60 mV or −30.−60 mV), VDAC1 shows diminished permeability to large anions and becomes more selective to small cations (72). However, it is unknown whether the voltage dependence of VDAC1 is relevant in physiological conditions, as the value of membrane potential across OMM is unknown. It is generally believed that any membrane potential generated at OMM will be offset by a relatively undisturbed movement of small ions across OMM. However, there is a theoretical possibility that OMM can be polarized to potentials large enough to alter the permeability of VDAC1 (2, 3). Although the role of OMM potential in the regulation of VDAC1 permeability is unlikely, it remains to be investigated whether potential across OMM changes in CRC and whether such change can alter Km(ADP).

#### Hexokinase-VDAC Interaction Regulates the Permeability of VDAC to Adenine Nucleotides

**Figure 2:**
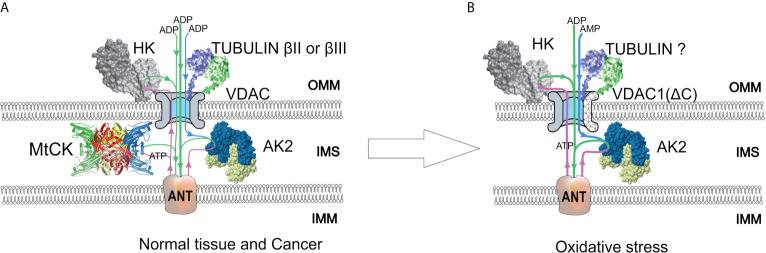
A model of regulation of outer mitochondrial membrane (OMM) permeability for adenine nucleotides in normal and colorectal cancer (CRC) cells. Voltage-dependent anion channel (VDAC) is the pore through which adenine nucleotides move into and out of the mitochondria. **(A)** In normal and possibly some cancer cells, a minor amount of hexokinase (HK) is bound to VDAC and utilizes mitochondrial ATP to initiate glycolysis. Produced ADP is channeled back to the mitochondrial matrix *via* VDAC and adenine nucleotide translocase (ANT) for use in oxidative phosphorylation (OXPHOS). VDAC permeability is also regulated by tubulin binding. As a result of beta-tubulin-VDAC interaction, the VDAC is less permeable to adenine nucleotides. This in turn promotes cells to use creatine kinase (CK) and adenylate kinase (AK) energy transfer networks for intracellular distribution of high-energy phosphates. Mitochondrial intermembrane space (IMS)-residing mitochondrial CK (MtCK) is functionally coupled to ANT, turning OXPHOS to be dependent on ADP originating from MtCK reaction. Mitochondrial AK isoform AK2 uses AMP passing through VDAC and ATP passing through ANT to produce ADP, which stimulates OXPHOS. These energy transport systems provide feedback between ATP consumption and synthesis. **(B)** Redox stress may induce an increased amount of HK bound to VDAC. In addition, VDAC can be truncated at C-terminus by proteases activated in response to oxidative stress. The role of tubulin in the regulation of VDAC permeability remains unclear, as the interaction of truncated VDAC with tubulin might be impaired. The AK2 activity in cancer cells is increased, resulting in enhanced utilization of extra-mitochondrial AMP to OXPHOS. IMM, inner mitochondrial membrane; IMS, intermembrane space.

Although the VDAC-hexokinase (HK) binding was demonstrated by several groups using different experimental approaches, it still remains somewhat speculative, and there are different hypothesis on its functional consequences. Research activities of Prof. Pedersen and his colleagues resulted in the discovery of the binding of HK-II to VDAC with the conclusion that this phenomenon could play a pivotal role in the “Warburg Effect” (76–80). Review paper of V. Shoshan-Barmatz et al. proposed the hypothesis that HK-II binds to VDAC and promotes VDAC closing (81). Neumann et al. demonstrated the binding of the cytosolic protein HK-I to VDAC by two-color STED microscopy (82). Our group showed the colocalization of VDAC1 and hexokinase II in cell cultures and clinical cancer samples by confocal microscopy imaging (6, 67). Based on these studies, two models of VDAC permeability control have been proposed. The model proposed by Pedersen et al. states that the binding of HK-II to VDAC plays a pivotal role in maintaining the Warburg phenotype in cancer cells (77, 83). In such a setting, mitochondrial ATP is preferentially directed to glycolysis (HK reaction) and the produced ADP is channeled back to the OXPHOS (**Figure 2**). At the same time, VDAC is assumed to be in an open state and mitochondria have free access to exogenous ADP (84, 85), thus low Km(ADP) values are expected. Glucose-stimulated increase of mitochondrial respiration shows the amount of ADP released in the HK reaction that passes through VDAC and is utilized in mitochondrial ATP synthesis (86). Such glucose effect comprises a fraction of total ADP-stimulated respiration and is higher in cancer cells as compared to normal cells. Accordingly, the glucose effect is about 20% for CRC tissue, about 12% for normal colon tissue samples (6), and about 48% for Caco-2 CRC cell line (43). These results show that the lower affinity of mitochondria for ADP could be related to the weaker ability for glucose to stimulate respiration. CRC displays elevated levels of VDAC1 as compared with surrounding healthy tissues (43), and this is in good agreement with the fact that V_max_ for ADP-dependent respiration is higher in CRC (44). The total HK activity and expression levels of HK1 and HK2 in CRC do not differ from that of normal tissue (6, 44). In both the normal mucosa and the CRC, HK2 is colocalized with VDAC (6, 43). The interaction of HK1 or HK2 with VDAC1 gives numerous advantages to cancer cells: (1) it mediates the increased permeability of the OMM to adenine nucleotides; (2) it increases the rate of aerobic glycolysis and thereby allows the cells to adapt to hypoxic conditions; (3) it mediates elevated resistance to apoptosis and protection from oxidative stress as VDAC1-bound HK acts as an anti-apoptotic protein (73, 87–89). VDAC-HK interaction is reversed with inhibitors of HK2 (e.g., 3-bromopyruvate), and agents that disrupt the VDAC-HK interaction have been tested as anticancer drugs (73, 90–93). It was also reported that silencing of VDAC1 expression by siRNA inhibited the proliferation of several cancer cell lines (including CRC) (94).

#### Free Beta-Tubulins Controlling VDAC Permeability in CRC

According to the free-tubulin model, the binding of free tubulin blocks VDAC and thereby regulates respiration (95). The rationale behind this model is the observation that proliferating cancer cells have high levels of free tubulin for mitotic spindle formation. Free tubulin dimers bound to VDAC induce a closed state of VDAC (**Figure 2**) and cause a suppression of mitochondrial metabolism; thus, aerobic glycolysis will become the main source of energy. Maldonado and Lemasters’s group shows at HepG2, A549, and UM-SCC-1 cells that tubulin binding closes the VDAC channel (95). It sounds like the hypothesis in this review contradicts Maldonado’s publications (95, 96). However, in fact, the results of both works are in agreement. The amount of dimeric and polymerized tubulin in cells is nearly constant, but the ratio could change significantly. In both cases it is dimeric tubulin, which affects VDAC permeability, but this effect depends on the polymerization state. Also, it should be definitely noted that the regulation of VDAC permeability is tissue specific. Unlike striated muscles, where the main regulator of VDAC is beta-II tubulin (97), in CRC the VDAC and beta-II tubulin colocalization is absent (6). Instead, beta-III tubulin (TUBB3) could be the partner of VDAC in CRC cells. Beta-III tubulin overexpression has been reported in several intestinal cancers like carcinoids of the small intestine and rectal carcinoids (98), gastric cancer (99), colon neoplasias like polyps, and CRC (6, 100). *TUBB3* expression has been associated with the resistance to drugs perturbing the microtubule dynamics (e.g., paclitaxel) and studied as a prognostic biomarker in various cancers (101, 102). It has been demonstrated that in non-small-cell lung cancer, the expression of beta-III tubulin decreases the dependence of cells on glycolysis and thus improves the tumor’s ability to cope with the changing nutrient supply in the microenvironment (103). From a functional analysis of the network of proteins forming disulfide bonds with beta-III tubulin, it appears that some of them are involved in oxidative stress and glucose deprivation response (104). It was shown that hypoxia *via* HIF-1α can induce the expression of *TUBB3* (105). Beta-III tubulin is likely part of a complex pathway induced by hypoxia and shortage of nutrients (101). However, our recent study revealed that microtubule destabilizing (colchicine) and stabilizing (taxol) agents do not affect the Km(ADP) in glioblastoma and sarcoma cells (67). Hence, the actual role of beta-tubulins in cancer metabolism and mitochondrial respiratory control needs further investigation.

#### Regulation of VDAC1 by Protein-Protein Interactions and Redox Stress

In addition to the two previous models, the modifications of VDAC1 protein induced by oxidative stress could be responsible for alterations of apparent value of Km(ADP). Tumor cells are well adapted to a hypoxic environment, and VDAC1 is regulated by oxygen tension in HIF-1α-dependent manner at the levels of transcription and protein modification. Transcription of the *VDAC1* gene is regulated by HIF-1α and NRF-1 (nuclear respiratory factor 1), which leads to increased levels of VDAC1 in response to hypoxia or nutrient deprivation of the cells (106). Along with VDAC1 expression regulation, HIF-1α is also involved in the cleavage of VDAC1, resulting in a truncated form of VDAC1 (107). In normoxic conditions, VDAC1 is expressed as a full-length protein of molecular weight of approximately 30 kDa, while in response to hypoxia, there is a larger proportion of a shorter VDAC1 variant lacking C-terminal part (VDAC1-ΔC) with a molecular weight of approximately 25 kDa (107). The shorter variant is a product of the cleavage of VDAC1 at asparagine 214 by the asparagine endopeptidase Legumain (LGMN), which in turn is activated in a HIF-1α-dependent way upon hypoxia (107). The electrophysiological properties of VDAC1-ΔC are similar to full-length protein; however, its permeability is slightly reduced (107). Levels of VDAC1-ΔC were higher in late-stage lung tumors (107), and it was suggested that HIF-1α mediated induction of VDAC1-ΔC provides protection from apoptosis and enhances cell survival in hypoxia (107, 108). Hypoxia-induced VDAC1-ΔC lacks a phosphorylation site at serine 215, and therefore its interaction with tubulin is impaired (108). Notably, *HIF-1α* overexpression was significantly associated with higher CRC-specific mortality in a cohort of 731 patients (109). Consequently, inhibition of HIF-1α is proposed as a possible treatment strategy for CRC (110). Moreover, the expression of endopeptidase LGMN is elevated in CRC and is associated with a poor prognosis (111). Furthermore, a meta-analysis revealed the overexpression of *LGMN* to be correlated with the aggressiveness of different cancer types, with higher levels of LGMN in late-stage tumors (112).

It is currently unknown whether VDAC1-ΔC is present in CRC cells and whether truncation-induced impairment of VDAC1 interaction with tubulin affects apparent affinity for ADP (**Figure 2**). Given the role of tubulin in the regulation of VDAC1 and the discovery of VDAC1-ΔC in lung cancer, VDAC1 truncation may also play a role in metabolic alterations of CRC. Future studies should reveal whether the truncated form of VDAC1 plays a role in metabolic adaptations of CRC.

Recent studies indicate a link between iron-sulfur cluster (ISC) synthesis and regulation of VDAC1. Biogenesis of ISC is an ancient process, and ISCs are important redox-sensitive cofactors for many enzymes involved in energy homeostasis. Synthesis of ISC starts within the mitochondrial matrix, and depletion of proteins involved in mitochondrial ISC assembly leads to accumulation of VDAC1-ΔC in normoxic conditions independent of HIF-1α (113). Depletion of the iron-sulfur cluster containing protein CISD2 also resulted in the accumulation of truncated VDAC1-ΔC (113). Therefore, mitochondria-associated membrane-localized Fe-S protein CISD2 acts as a link between ISC machinery and accumulation of VDAC1-ΔC (113).

Another iron-sulfur cluster protein, mitoNEET, was found to interact with VDAC1 in a redox-sensitive way (114). MitoNEET harbors [2Fe-2S] cluster and binds to VDAC1 when its cluster is oxidized, thus inhibiting VDAC1 conductivity. Such interaction does not occur when mitoNEET-bound ISC cluster is reduced (114). Therefore, mitoNEET governs VDAC1 permeability in a redox-sensitive way, inhibiting VDAC1 in high redox stress conditions. Oxidative stress is increased in CRC (115); thus, the interaction of mitoNEET with VDAC1 can be altered in CRC. It remains to be investigated whether such redox-sensitive mitoNEET-VDAC1 interaction can alter the apparent Km(ADP) value and is involved in the metabolic plasticity of CRC.

There is a large number of proteins that were found to interact with VDAC1 and are therefore potentially able to modulate VDAC permeability. Interacting partners of VDAC1 are involved in the regulation of apoptosis (Bax, Bcl2, Bak, etc.), energy metabolism (HK1, HK2, ACSL, CPT1, ANT, etc.), cytoskeletal organization (Tubulin, actin, dynein, etc.), and other cellular functions [Parkin, alpha-synuclein, APP, gamma-secretase) [reviewed in (116)]. However, the role of these interactions in the modulation of cellular respiration needs to be further investigated.

## Energy Transport Pathways in CRC Cells—The Participants in the Metabolic Plasticity

In addition to the altered transport of adenine nucleotides through OMM alterations of energy transport circuits formed from creatine kinase (CK) and adenylate kinase (AK) isoenzymes are also involved in the development of metabolic plasticity. Cancer cells have uncontrolled cell division, which is accompanied by a high energy need for anabolic processes and large cell structure rearrangements. Therefore, it is hypothesized that energy transport pathways are also reprogrammed in cancer cells to meet these demands. Previous data show downregulation of the CK pathway and mitochondrial CK (MtCK) in CRC cells, which results in functional uncoupling between the CK circuit and OXPHOS (6, 44). In contrast, total AK activity is higher in CRC than in normal intestinal tissue, and it also reflects enhanced coupling between AK and OXPHOS (i.e., AMP can affect the rate of oxygen consumption) (**Figure 2**) (6, 44). This is in agreement with the observation that expression of AK mitochondrial isoform AK2 is increased in several cancers including lung adenocarcinoma (117) and breast cancer (118, 119). Also, there is evidence that another mitochondrial isoform, AK4, is involved in the regulation of mitochondrial metabolism in cancer cells. In HeLa cells, AK4 forms complexes with ANT, VDAC, and HK2 for the efficient recycling of ADP (120). Further, AK4 expression is induced by hypoxia, and protein complex AK4-ANT-VDAC-HK2 complex supports the high glycolytic activity of cancer cells (120). Intestinal cells are able to switch off the CK circuit and turn on the AK pathway to establish metabolic plasticity. Such flexibility of phosphotransfer networks in Caco2 CRC cell lines depends on the availability of key metabolic substrates and is associated with the cell differentiation state (121). The abovementioned data indicate a possible role of the phosphotransfer networks related to the regulation of VDAC permeability for adenine nucleotides and metabolic plasticity.

The function of energy transfer pathways is well characterized in striated muscle cells where its role is to overcome the diffusion restrictions for ATP and ADP, thereby directing the energy-rich phosphate groups to the CK, AK, and glycolytic energy transfer circuits. This way of energy transfer allows the formation of micro-compartments at energy consumption sites where high ATP/ADP levels are maintained for maximal performance. Similarly, in the compartment where energy is produced (e.g., mitochondrial membranes), favorable levels of ADP are maintained to ensure efficient ATP synthesis [reviewed in (65, 122)]. In the case of CRC, downregulation of MtCK leads to the inability to produce phosphocreatine and a loss of functional coupling between the VDAC-MtCK-ANT complex, accompanied by the formation of other regulating combinations like VDAC-HK-ANT. In this aspect, more studies are required to determine the profile of HK, AK, ANT, and VDAC isoform expression in human CRC.

In addition to their role in energy transfer among cellular processes, AKs are an integral part of intracellular energy sensing and metabolic signaling (123, 124). Due to its catalytic reaction (2ADP ↔ AMP + ATP), it can amplify a small change in the ATP/ADP ratio into relatively large changes in AMP concentration. This relates AKs to the activation of cellular AMP-sensitive components like AMPK. In general, activation of AMPK switches on catabolic pathways that generate ATP, while switching off biosynthetic pathways and cell-cycle progress (125). The role of AMPK in cancer is controversial; it has been recognized as a tumor suppressor in some cancers (126–129) and in some cases described as a contextual oncogene, as the AMPK activation promotes tumor progression and chemoresistance (130–132). Downregulation of AK→ AMP→ AMPK signaling could lead to loss of control over the cell cycle, growth, and proliferation (124). A recent in-depth review about AKs and metabolic signaling in cancer cells by Klepinin et al. (124) highlights the role of suppression of AK phosphotransfer and signaling through AMPK as a potential target for cancer metabolism. How different AK isoforms are distributed in CRC cells and how their activities affect AMPK activation and metabolic plasticity need further investigation.

Adenylate kinases network promotes cancer growth and metastasis through participating in AMPK metabolic signaling and regulating mitochondrial adenine nucleotide exchange.

## Conclusion and Prospects

Metabolic plasticity is a defining characteristic of the cancer cells that allow undisturbed proliferation in changing environment. At the functional level, different metabolic states of the cancer cells can be identified and characterized by measuring the dependence of mitochondrial respiration upon ADP concentration using the classical Michaelis-Menten kinetic model. The apparent affinity of ADP provides an integrated assessment of cell metabolic state, which is functionally determined by the permeability of VDAC1. Regulation of VDAC1 involves many protein-protein interactions, as well as hypoxia- and redox-sensitive mechanisms. The regulation of OMM permeability for adenine nucleotides is presumably more complex than the binding between the VDAC1 channel and some single type of protein molecule. Unraveling the molecular mechanisms of metabolic plasticity will reveal new therapeutic targets for the development of novel cancer treatments. This knowledge combined with relatively simple functional evaluation of cancer metabolism in biopsy material can form a new prospect for personalized medicine.

## Author Contributions

Conceptualization, LT, MP, AT, and TK. Funding acquisition, TK. Project administration, AT and TK. Visualization, LR and IS. Writing—original draft, MP, AT, VC, and TK. Writing—review and editing, LR, SM, ER-K, NT, KT, IS, and TK. All authors contributed to the article and approved the submitted version.

## Funding

This work was supported by the Estonian Research Council grant PRG1035 and NICPB institutional Development Fund grant.

## Conflict of Interest

The authors declare that the research was conducted in the absence of any commercial or financial relationships that could be construed as a potential conflict of interest.

## Publisher’s Note

All claims expressed in this article are solely those of the authors and do not necessarily represent those of their affiliated organizations, or those of the publisher, the editors and the reviewers. Any product that may be evaluated in this article, or claim that may be made by its manufacturer, is not guaranteed or endorsed by the publisher.

## Abbreviations

ADP, adenosine diphosphate; AMPK, adenosine 5’-monophosphate-activated protein kinase; AK, adenylate kinase; ANT, adenine nucleotide translocator; CK, creatine kinase; CRC, colorectal cancer; HK, hexokinase; HIF, hypoxia-inducible factor; ISC, iron-sulfur clusters; OMM, outer mitochondrial membrane; TCA, tricarboxylic acid; OXPHOS, oxidative phosphorylation; ROS, reactive oxygen species; VDAC, voltage-dependent anion channel.
